# Elevated Atmospheric CO_2_ and Nitrogen Fertilization Affect the Abundance and Community Structure of Rice Root-Associated Nitrogen-Fixing Bacteria

**DOI:** 10.3389/fmicb.2021.628108

**Published:** 2021-04-21

**Authors:** Jumei Liu, Jingjing Han, Chunwu Zhu, Weiwei Cao, Ying Luo, Meng Zhang, Shaohua Zhang, Zhongjun Jia, Ruihong Yu, Ji Zhao, Zhihua Bao

**Affiliations:** ^1^Ministry of Education Key Laboratory of Ecology and Resource Use of the Mongolian Plateau and Inner Mongolia Key Laboratory of Grassland Ecology, School of Ecology and Environment, Inner Mongolia University, Hohhot, China; ^2^Inner Mongolia Key Laboratory of Environmental Pollution Control and Waste Resource Reuse, Inner Mongolia University, Hohhot, China; ^3^Chongqing Key Laboratory of Environmental Materials and Remediation Technologies, College of Chemistry and Environmental Engineering, Chongqing University of Arts and Sciences, Chongqing, China; ^4^State Key Laboratory of Soil and Sustainable Agriculture, Institute of Soil Science, Chinese Academy of Sciences, Nanjing, China

**Keywords:** elevated atmospheric CO_2_, nitrogen fertilization, rice paddy, plant-associated nitrogen-fixing bacteria, growth stages

## Abstract

Elevated atmospheric CO_2_ (eCO_2_) results in plant growth and N limitation, yet how root-associated nitrogen-fixing bacterial communities respond to increasing atmospheric CO_2_ and nitrogen fertilization (eN) during the growth stages of rice is unclear. Using the *nifH* gene as a molecular marker, we studied the combined effect of eCO_2_ and eN on the diazotrophic community and abundance at two growth stages in rice (tillering, TI and heading, HI). Quantitative polymerase chain reaction (qPCR) showed that eN had no obvious effect on *nifH* abundance in rice roots under either ambient CO_2_ (aCO_2_) or eCO_2_ treatment at the TI stage; in contrast, at the HI, *nifH* copy numbers were increased under eCO_2_ and decreased under aCO_2_. For rhizosphere soils, eN significantly reduced the abundance of *nifH* under both aCO_2_ and eCO_2_ treatment at the HI stage. Elevated CO_2_ significantly increased the *nifH* abundance in rice roots and rhizosphere soils with nitrogen fertilization, but had no obvious effect without N addition at the HI stage. There was a significant interaction [CO_2_ × N fertilization] effect on *nifH* abundance in root zone at the HI stage. In addition, the *nifH* copy numbers in rice roots were significantly higher at the HI stage than at the TI stage. Sequencing analysis indicated that the root-associated diazotrophic community structure tended to cluster according to the nitrogen fertilization treatment and that *Rhizobiales* were the dominant diazotrophs in all root samples at the HI stage. Additionally, nitrogen fertilization significantly increased the relative abundance of *Methylosinus* (*Methylocystaceae*) under eCO_2_ treatment, but significantly decreased the relative abundance of *Rhizobium* (*Rhizobiaceae*) under aCO_2_ treatment. Overall, the combined effect of eN and eCO_2_ stimulates root-associated diazotrophic methane-oxidizing bacteria while inhibits heterotrophic diazotrophs.

## Introduction

Rice is the most important staple food for half the world’s population, and nearly 90% of the rice fields in the world are located in Asia (Food and Agriculture Organization of the United Nations, 2002). Rice fields act as an important carbon and nitrogen cycling interface between the atmosphere and land (Ishii et al., 2011; Tokida et al., 2011). Carbon dioxide (CO_2_) fixation by photosynthesis provides energy for rice plants. Atmospheric CO_2_ concentrations have increased from approximately 270–400 ppm by human activity since preindustrial times (IPCC, 2013). Elevated concentrations of atmospheric CO_2_ under a free-air CO_2_ enrichment (FACE) system could increase the biomass, yield and grain quality of rice plants (Usui et al., 2014; Zhu et al., 2015). Elevated CO_2_ (eCO_2_) can variously influence underground nutrient cycling, which is mainly mediated by microbial communities (García-Palacios et al., 2015). For example, CH_4_ emissions were significantly increased by eCO_2_ and/or temperatures in rice paddies (Tokida et al., 2011; Xie et al., 2012) due to the effects of methanogens and methanotrophs (Das and Adhya, 2012). CH_4_ generated in soil diffuses into rice roots, transported to the shoot via aerenchyma, and finally released from micropores in the leaf sheaths (Nouchi et al., 1990). The root zone (rice root and rhizosphere soil) was previously shown to be the main area CH_4_ oxidation in rice paddies (Bosse and Frenzel, 1997). A previous study reported that CH_4_ oxidation and N_2_ fixation were simultaneously activated in the root zone (root and rhizosphere soil) of wild-type rice in the low nitrogen field (Bao et al., 2014b). Further, ^15^N-N_2_ feeding experiments and metaproteomics analysis demonstrated that *Methylosinus* was a core diazotroph in rice roots and likely contributed to both CH_4_ oxidation and N_2_ fixation processes (Bao et al., 2014a; Shinoda et al., 2019). *Bradyrhizobium* and/or *Methylosinus* were frequently detected in rice roots using metagenomic analysis (Eller and Frenzel, 2001; Ikeda et al., 2014), and they were affected by the rice growth stage under eCO_2_, based on 16S rRNA gene analysis (Okubo et al., 2014). eCO_2_ quantitatively changed the release of labile sugars, organic acids, and amino acids from plant roots (Bhattacharyya et al., 2013), possibly influencing the activity of rhizospheric and root-associated microbes.

In rice production, nitrogen is often a limiting factor. Supplementation with N fertilizer has increased rice grain yields over recent decades (Yang et al., 2006). However, the heavy use of N fertilizers causes various environmental impacts, such as nitrous oxide emissions, soil and water body N deposition, reactive N leaching, eutrophication and methane emissions (Ju et al., 2009). Thus, the impacts of nitrogen input on methane emissions and methane cycling bacteria, including methanotrophs and methanogens, have attracted worldwide attention (Shrestha et al., 2010; Banger et al., 2012; Bao et al., 2014a,b). N fertilization decreased the relative abundance of root-associated bacteria community, especially type II methanotrophs and *Bradyrhizobium* (Ikeda et al., 2014). Generally, N fertilizer inputs inhibit N_2_ fixation and influence the N_2_-fixing bacterial community (Kumar et al., 2018), which can convert atmospheric N_2_ gas to ammonium and make it available to plants as a major nutrient (Yoneyama et al., 2019). eCO_2_ can increase the N demand for plants, which is beneficial for N_2_ fixation (Luo et al., 2004). A previous study showed that soil diazotrophic abundance significantly increased in two difference rice cultivars to satisfy the increased N demand under eCO_2_ in paddy fields (Yu et al., 2018). This may be due to an increase in the organic acid content of the root system released into the soil by eCO_2_ (Bhattacharyya et al., 2013), thus providing carbon source and energy to N_2_-fixing bacteria. However, plant roots associate with diverse soil-derived microbes including N_2_-fixing bacteria, which influences plant nutrient uptake (Berendsen et al., 2012; Shinoda et al., 2019). In addition, root-associated microorganism significantly changed between different growth stages in rice paddy field (Chaparro et al., 2014; Okubo et al., 2014). Whether the abundance and community composition of N_2_-fixing bacteria in the rhizosphere soils and roots of rice are affected by eCO_2_ and nitrogen fertilization at two different stages during the rice-growing season is unclear, as is which nitrogen fixers respond to these factors.

Using qPCR and MiSeq sequencing techniques, we studied the abundance and community composition of N_2_-fixing bacteria in the roots of rice plants (*japonica* rice, WuYunJing) from a free-air CO_2_ enrichment (FACE) system in Yangzhou city, Jiangsu, China. In this study, we (i) examined the combined effects of eCO_2_ and N fertilization on the community composition of N_2_-fixing bacteria in the rice roots and/or rhizosphere at the tillering and heading stages and (ii) identified the N_2_-fixing bacteria responsive to the treatments. The results will be valuable for understanding the strategies by which root-associated N_2_-fixing bacterial communities form to adapt to prospective climatic changes.

## Materials and Methods

### Study Site and Experimental Design

**FIGURE 1:**
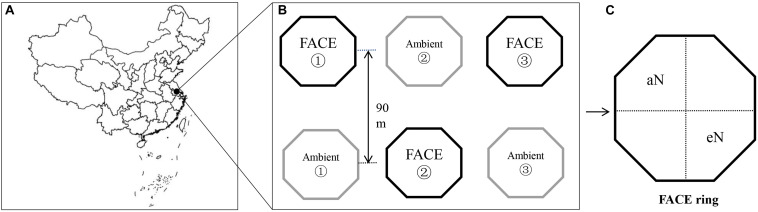
Sampling site diagram of *japonica* rice WuYunJing from the free-air CO_2_ enrichment (FACE) system **(B,C)** in Yangzhou city, Jiangsu, China (119°42′0″E, 32°35′5″N) **(A)**.

The experimental FACE platform was located in Zongcun Village (119°42′0′E, 32°35′5″N), Yangzhou City, Jiangsu Province (Figure 1; Yu et al., 2018). The long-term experimental platform with a rice-wheat rotation crop system started in 2004. From 2010, the rice-wheat rotation system was changed to a rice-fallow system (Yu et al., 2018). The region has a north subtropical monsoon climate with a mean annual temperature of 16°C and a mean annual precipitation of 900–1,000 mm. The experiment had a split-plot design, with CO_2_ as the main factor and N fertilization and rice cultivar as the split-plot factors. More details about the FACE system are provided in by Zhu et al. (2016). In brief, three rectangular paddy fields were selected for their uniformity in growth and yield for use in the experiment. Within each field, a FACE plot was paired with an ambient control, and the plot centers were 90 m apart to avoid cross-contamination by CO_2_ (Heim et al., 2009; Figure 1). The CO_2_ levels of each FACE ring were maintained with at ambient CO_2_ (aCO_2_, 400 ± 10 μmol mol-1) or eCO_2_ (590 ± 40 μmol mol-1) concentrations corresponding to the future expectations of the Intergovernmental Panel on Climate Change (IPCC, 2013). Each FACE plot was encircled with an octagonal ring (12.5 m in diameter) equipped with emission tubes surrounding the crops that injected pure CO_2_ at approximately 50 cm above the crop canopy throughout the day. The target CO_2_ concentration within the FACE rings was controlled by a real-time CO_2_ monitoring system (Figure 1). The aCO_2_ rings did not receive any supplemental CO_2_.

There were two N fertilization levels, no N fertilization (aN) and elevated N fertilization (eN) (Figure 1). The eN plots were fertilized with N, P, and K (in the form of N, P_2_O_5_, and K_2_O). In the aN plots, only P and K were applied (in the form of P_2_O_5_ and K_2_O, respectively). P (90 kg ha^–1^) and K (90 kg ha^–1^) were applied as basal fertilizers to both aN and eN rice field plots before transplanting. The total nitrogen fertilization rate was 225 kg ha^–1^, with 40, 30, and 30% of the total amount applied to only eN rice field plots before transplanting, tillering, and heading, respectively (Zhu et al., 2016). The soil in the present study is a Shajiang-Aquic Cambisol soil and has a sandy loam texture (13.7% clay, 28.5% silt, and 57.8% sand). At the beginning of the study, the initial soil properties were analyzed and recorded as follows: soil organic carbon (17.9–18.4 g kg^–1^ in aCO_2_ plots and 18.5–18.6 g kg^–1^ in eCO_2_ plots), total N (1.6–1.7 g kg^–1^ in aCO_2_ plots and 2.01–2.07 g kg^–1^ in eCO_2_ plots), total P (0.6 g kg^–1^ in aCO_2_ plot and 0.7 g kg^–1^ in eCO_2_ plot), total K (13.8 g kg^–1^ in aCO_2_ plot and 14.3 g kg^–1^ in eCO_2_ plot), available P (8.6 mg kg^–1^ in aCO_2_ plot and 11.7 mg kg^–1^ in eCO_2_ plot), and available K (66.0 mg kg^–1^ in aCO_2_ plot and 75.0 mg kg^–1^ in eCO_2_ plot) (see Supplementary Table 1 in the Supplementary Material).

### Rice Plant and Soil Sampling

Rice plants (*japonica*, WuYunJing) with soil blocks were sampled from the aCO_2_ rings and eCO_2_ rings. Three plants per experimental plot were harvested on 21 July 2014 (30 d after transplanting, [DAT]), which corresponded to the TI stage (the vegetative stage of rice), and on 25 August 2014 (65 d DAT), which corresponded to the HI stage (the reproductive stage of rice). The samples were immediately transported on ice to the laboratory. The rice roots and rhizosphere soils were sampled as described in previous studies within 1 day after field sampling (Kimura, 2004; Bao et al., 2014a). Briefly, after the rice plants with soil blocks were sampled, the soil blocks, including rice plants, were divided vertically into two equal parts for root collection. Some of the exposed roots were carefully picked from the plants using sterilized forceps and placed into a 50 mL centrifuge tube containing sterile water. The roots were also washed by centrifugation before and after sonication (90 watts for 5–10 min) and the three pellets were combined to form the rhizosphere soil samples. After the rhizosphere soil was removed, the rice root samples were transferred to new centrifuge tubes (50 mL) containing sterile water and centrifuged at 4°C for 10 min at 8,000 × *g*; the pellet from this centrifugation was considered the root sample. At two different growth stages, a total of 24 root and 24 rhizosphere soil samples were obtained and stored at –80°C, and molecular analysis was conducted within 6 months of sampling.

### DNA Extraction and Quantification of nifH Genes

Genomic DNA was extracted from root and rhizosphere soil samples using the Fast DNA SPIN Kit for Soil (MP Biomedicals, Solon, OH, United States) according to the manufacturer’s protocol. For root samples, the frozen tissues were ground into powder using a mortar and pestle under liquid nitrogen before DNA extraction. The extracted DNA was immediately stored at –20°C after measurement of the DNA concentration.

The abundance of nitrogen fixers was quantified by qPCR targeting of the *nifH* gene from 24 root and 24 rhizosphere soil samples, respectively. The reactions were performed on a CFX Connect Optical Real-Time Detection System (Bio-Rad Laboratories, Hercules, CA, United States) in volumes of 20 μL and containing 1 μL extracted DNA (approximately 50 ng), 10 μL 2 × SYBR Premix Ex Taq (Takara Biotech, Dalian, China), and 500 nM primers PolF (Poly et al., 2001) and AQER (Wartiainen et al., 2008). The parameters were those previously described in the literature (Yu et al., 2018). Briefly, the amplifications were performed with an initial denaturation step at 95°C for 30 s, followed by 36 cycles of denaturation at 95°C for 30 s, annealing at 57°C for 45 s and extension at 72°C for 45 s. The specificity of the amplification products was confirmed by melting curve analysis, and the expected sizes (340 bp) of the amplified fragments were checked in a 1.5% agarose gel stained with ethidium bromide.

Standard curves were obtained using 10-fold serial dilutions of the linear *Escherichia coli*-derived vector plasmid pGEM-T Easy Vector (Promega, Madison, WI) containing a cloned *nifH* gene derived from *Bradyrhizobium diazoefficiens* USDA 110 (NZ_CP011360). The concentration of the plasmid DNA was measured by a NanoPhotometer P-Class P-330C (IMPLEN, Munich, Germany) and used for the calculation of standard copy numbers. The *R*^2^-values and amplification efficiencies were as follows: *R*^2^ = 0.996–1.000, efficiency = 94.0–97.0%. Melting curve analysis was used to confirm the specific amplification of target genes and always showed a single peak.

### MiSeq Sequencing of nifH Gene Amplicons

The *nifH* gene was amplified using PCR primers PolF (TGC GAY CCS AAR GCB GAC TC) and AQER (GAC GAT GTA GAT YTC CTG) (Poly et al., 2001; Wartiainen et al., 2008), which was used to detected N_2_-fixing bacteria including *Alphaproteobacteria*, *Betaproteobaceria*, *Gamaproteobacteria*, and *Firmicutes* in paddy field (Yu et al., 2018). A unique 12-bp barcode was added to each sample at the 5′-end of the reverse primer. The 20 mL reaction mixtures included 4.0 μL 5 × FastPfu Buffer (plus Mg^2+^), 2.0 μL 2.5 mM each dNTP, 0.4 μL 5 U/μL TransStart Fastpfu DNA Polymerase (TransGen Biotech, Beijing, China), 0.8 μL 5 μM each primer, 0.2 μL 20 mg mL^–1^ bovine serum albumin (BSA; Amesco, Tampa, United States) and 10 ng template DNA. The amplification conditions were as follows: initial denaturation at 95°C for 15 min, followed by 36 cycles of 94°C for 1 min, 55°C for 1 min, elongation at 72°C for 1 min, and a final extension step of 72°C for 10 min. The amplification products were approximately 340 bp. Each sample was amplified with three technical replicates, purified with an AxyPrep DNA Gel Extraction Kit (Axygen Biosciences, Union City, CA, United States) and quantified using QuantiFluor^TM^-ST (Promega, United States) according to the manufacturer’s protocol. Purified amplicons were pooled in equimolar amounts and paired-end sequenced (2 × 300) on an Illumina MiSeq platform (Illumina, San Diego, United States) according to the standard protocols by Majorbio Bio-Pharm Technology Co., Ltd. (Shanghai, China). In order to determine what kind of N_2_-fixing bacteria was present in a large number at the heading stage, we focused on the heading stage and examined the diversity and composition of the diazotrophic community at this stage. 12 root samples at the heading stage were used for sequencing. The raw sequences (fastq files) of the 12 rice root samples at the heading stage were deposited in the Sequence Read Archive (SRA) of NCBI under accession numbers SRR10321589–SRR10321600.

### Processing of the Sequencing Data

Raw sequences were analyzed by QIIME software (Caporaso et al., 2010). All sequence reads were trimmed and assigned to each sample based on their barcodes, and then quality filtering was performed. Sequences less than 150 bp in length were removed. Chimera removal was carried out using the software program Mothur (Schloss et al., 2009). The nucleotide sequences of *nifH* were further converted to amino acid sequences using the FunGene Pipeline of the Ribosomal Database Project (Wang et al., 2013). The sequences encoding proteins that did not match the *nifH* protein sequence or that contained termination codons were discarded. The remaining sequences were aligned against the *nifH* gene database (Gaby and Buckley, 2014). The remaining high-quality sequences were clustered into operational taxonomic units (OTUs) at 90, 94, and 99% similarity, respectively, with UCLUST (Edgar, 2010; Berthrong et al., 2014), and they were used to estimate alpha diversity indices (e.g., coverage, observed species, Shannon and Simpson indices) by Mothur (Schloss et al., 2009). Taxonomic identity of the OTU clustering at 94% similarity was carried out by mapping representative OTU sequences to reference *nifH* sequences at the BLAST algorithm-based search site within GenBank. Finally, translated amino acid sequences of each representative of top 30 OTUs were aligned by using the CLUSTAL W program (Thompson et al., 1994), and neighbor-joining phylogenetic tree was constructed by using MEGA version 5 (Tamura et al., 2011). Principal component analysis (PCA) were built using “vegan” package in *R* (version 3.1.2).

### Statistical Analysis

The experiment design was a split-plot factor arranged within a randomized complete block design with three replications (three rectangular paddy fields). The variance analyses were performed using linear mixed-effects model to test the effect of elevated CO_2_, N fertilization, and their interactions on *nifH* copy numbers, alpha diversity index (OTU numbers, Shannon and 1/Simpson), and the relative abundance of the main OTUs at the same growth stage. In these models, CO_2_ was treated as the fixed-effect whole-plot factor, N fertilization as the sub-plot factor, and block as the random effect factor. These analyses were performed in *R* (version 3.1.2). Multiple comparison was further used to determine which groups were significantly different using *Duncan* test in SPSS software, version 19.0 (IBM, Armonk, NY, United States). The comparison of *nifH* copy numbers at the tillering and heading stages were carried out by paired sample *t*-test procedure in SPSS software. Beta diversity was analyzed using permutational multivariate analysis of variance (PERMANOVA) to assess group differences in *R* (version 3.1.2). The significance level was *p* ≤ 0.05, while 0.05 < *p* ≤ 0.1 were considered marginally significant. The data are expressed as the means ± standard deviation.

## Results

### Effect of Elevated CO_2_ and N Fertilization on nifH Gene Abundance in the Root Zone at Different Growth Stages of Rice

**FIGURE 2:**
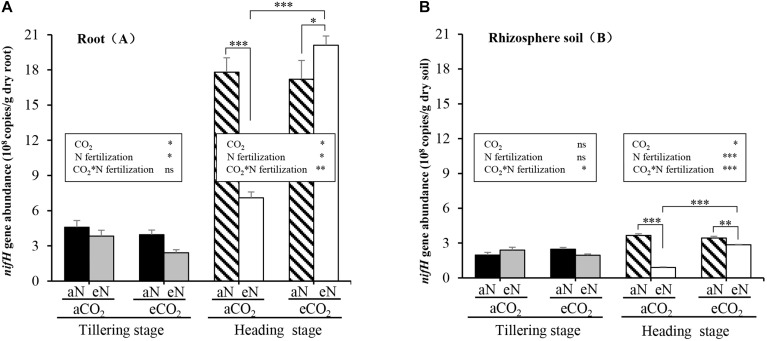
Interactive effects of elevated CO_2_ and N fertilization on *nifH* abundance in roots **(A)** and rhizosphere soils **(B)** of rice grown in the paddy field at the tillering (TI) and heading (HI) stages. aCO_2_, ambient CO_2_; eCO_2_, elevated atmospheric CO_2_. aN, no N fertilization; eN, elevated N fertilization. Considering the split-plot design, the statistics were derived using linear mixed-effects model procedure to test the effect of elevated CO_2_, N fertilization, and their interactions on *nifH* copy numbers at the same growth stage. Multiple comparison were further used to determine which groups were significantly different with *Duncan* test. The comparison of *nifH* copy numbers at the tillering and heading stages from the same plot with paired samples *t*-test (Data were showed in Supplementary Table 2). ^∗∗∗^*p* ≤ 0.001, ^∗∗^*p* ≤ 0.01, ^∗^*p* ≤ 0.05, ns *p* > 0.10.

To estimate the population sizes of N_2_-fixing bacteria in the root zone at different rice growth stages, we performed qPCR assays of roots and rhizosphere soils sampled from rice grown in the paddy field at the TI stage and at the HI stage. Firstly, we examined the effects of different growth stages on the abundance of N_2_-fixing bacteria. The copy numbers (× 10^8^ g ⋅ dry weight) of *nifH* genes in rice roots at the HI stage were significantly higher than those at the TI stage (*t* = 10.462, *p* = 0.009; *t* = 18.862, *p* = 0.003; *t* = 15.225, *p* = 0.004; *t* = 31.343, *p* = 0.001) (Supplementary Table 2), with the average increased by ∼4-fold (Figure 2A). Similarly, for rhizosphere soil samples, compared to the TI stage, the copy numbers of *nifH* genes were greater at the HI stage (*t* = 8.401, *p* = 0.014; *t* = 4.381, *p* = 0.048; *t* = 11.329, *p* = 0.008) (Supplementary Table 2), but only decreased under the combination of aCO_2_ and eN (*t* = −7.684, *p* = 0.017) (Supplementary Table 2 and Figure 2B).

Secondly, we examined the effects of elevated CO_2_, N fertilization, and their interaction on *nifH* copy numbers. Significant effects of elevated CO_2_ [*F*_(1, 2)_ = 37.4, *p* = 0.026], N fertilization [*F*_(1, 4)_ = 7.896, *p* = 0.048], but no significant interaction [CO_2_ × N fertilization] [*F*_(1, 4)_ = 0.877, *p* = 0.402] on *nifH* abundance in rice roots at the TI stage were observed (Figure 2A). Meanwhile, significant effects of elevated CO_2_ [*F*_(1, 2)_ = 47.52, *p* = 0.020], N fertilization [*F*_(1, 4)_ = 18.80, *p* = 0.012] and interaction [CO_2_ × N fertilization] [*F*_(1, 4)_ = 57.95, *p* = 0.002] on *nifH* abundance in rice roots at the HI stage were observed (Figure 2A). Further analyses showed that, at the HI stage, N fertilization reduced the abundance of *nifH* gene copies 2.5-fold (*p* < 0.001) under aCO_2_ but increased the abundance of *nifH* gene copies 1.2-fold (*p* = 0.031) under eCO_2_ (Figure 2A). Elevated CO_2_ doubled the copy numbers of *nifH* in rice root with N fertilization (*p* < 0.001), but had no significant effect (*p* = 0.607) without N addition at the HI stage (Figure 2A). Similarly, for rhizosphere soil samples, a significant interaction [CO_2_ × N fertilization] on *nifH* abundance either at the TI stage [*F*_(1, 4)_ = 15.21, *p* = 0.018] or the HI stage [*F*_(1, 4)_ = 188.7, *p* < 0.001] was observed, but significant effects of elevated CO_2_ [*F*_(1, 2)_ = 59.14, *p* = 0.017], N fertilization [*F*_(1, 4)_ = 450.9, *p* < 0.001] on *nifH* abundance were observed only at the HI stage (Figure 2B). And N fertilization significantly reduced the abundance of the *nifH* gene under either aCO_2_ (*p* < 0.001) or eCO_2_ (*p* = 0.001) treatment at the HI stage (Figure 2B). Elevated CO_2_ also significantly increased the copy numbers of *nifH* in rice rhizosphere soils with N fertilization (*p* < 0.001), but had no obvious effect without N addition (*p* = 0.109) at the HI stage (Figure 2B). As explained above, the combination of elevated CO_2_ and N fertilization has a significant effect on *nifH* abundance in rice zone (root and rhizosphere soil) at the HI stage (*p* < 0.05) (Figures 2A,B).

These results suggested that rice plants have the ability to enrich N_2_-fixing bacteria according to the growing season. Plants set the conditions for the proliferation of more N_2_-fixing bacteria at the HI stage than at the TI stage, and the combined effects of eCO_2_ and eN stimulate the abundance of N_2_-fixing bacteria in roots at the HI stage.

### Overview of N_2_-fixing Bacterial Community Structures in Rice Roots at the Heading Stage

The statistics from the high-throughput sequencing are summarized in Supplementary Table 3. *nifH* gene sequencing was performed on 12 rice roots DNA at the HI stage. After averaging each of the three parallel datasets, 47,849 high-quality reads were obtained, with 12,890, 10,998, 12,594, and 11,367 total *nifH* sequences for the aCO_2_-aN, aCO_2_-eN, eCO_2_-aN, and eCO_2_-eN plots, respectively. No significant effects of elevated CO_2_, N fertilization, and interaction [CO_2_ × N fertilization] on alpha diversity index (OTU numbers, Shannon and 1/Simpson) at the OTU cutoff values of 99, 94, and 90%, respectively, were found (*p* > 0.05) (data were showed in Supplementary Table 3). At the 99% OTU level, the coverage was 97% for the four plots. The coverage (> 99%) was high for libraries from each plot when OTUs were binned at 94% similarity (Supplementary Table 3). Therefore, we constructed a phylogenetic tree and performed taxonomic classification at 94% OTU similarity in this study.

**FIGURE 3:**
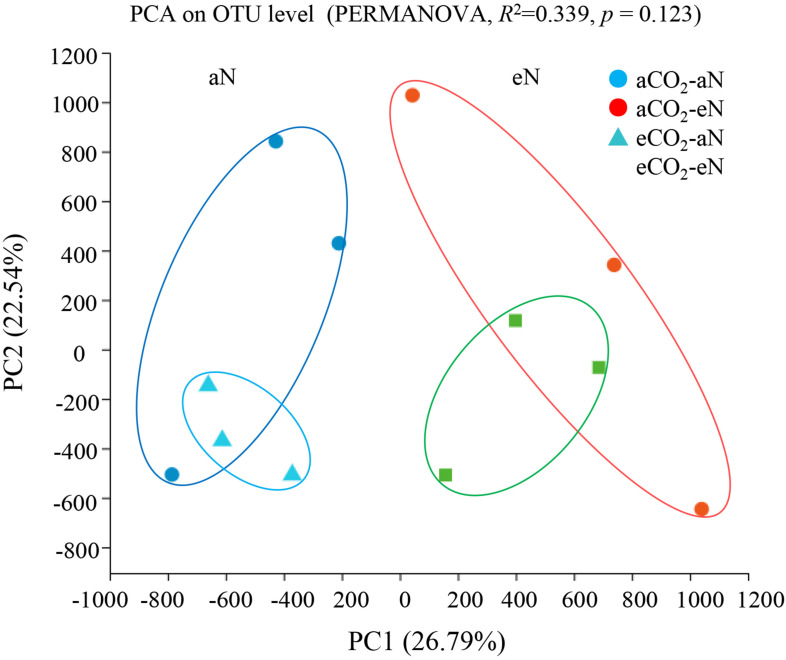
Principal component analysis (PCA) of *nifH* sequences at the 94% similarity OTU threshold by permutational multivariate analysis of variance (PERMANOVA) test. aCO_2_, ambient CO_2_; eCO_2_, elevated atmospheric CO_2_. aN, no N fertilization; eN, elevated N fertilization.

Principal component analysis (PCA) was performed using all sequence data (Figure 3) to obtain an overview of N_2_-fixing bacterial community shifts caused by elevated CO_2_, N fertilization, and their interaction. The results showed a cluster of community structures within the same N fertilization treatment, whereas no distinct separation was observed between two CO_2_ treatments (PERMANOVA, *p* = 0.123) (Figure 3).

### Compositions of N_2_-Fixing Bacterial Communities of Rice Roots at the Heading Stage

**FIGURE 4:**
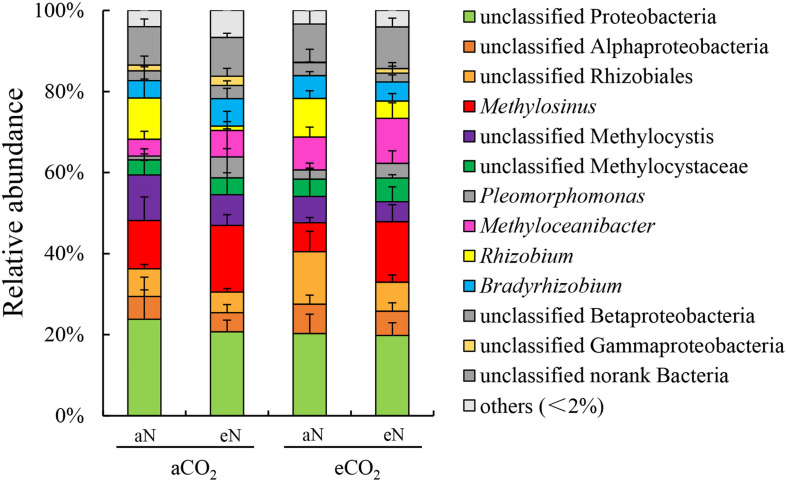
Relative abundance, at the genus level, of N_2_-fixing bacterial communities of rice roots at the heading stage under elevated CO_2_ and nitrogen fertilization treatments. aCO_2_, ambient CO_2_; eCO_2_, elevated atmospheric CO_2_. aN, no N fertilization; eN, elevated N fertilization.

The community compositions of root-associated N_2_-fixing bacteria were analyzed. Together at the order and family level, the rice root-associated N_2_-fixing bacterial communities were dominated by *Proteobacteria* (including unclassified *Proteobacteria, unclassified Alphaproteobacteria, unclassified Betaproteobacteria, unclassified Gammaproteobacteria*) (37.5–40.2%) and *Rhizobiales* (including unclassified *Rhizobiales, Methylocystaceae, Rhizobiaceae, Bradyrhizobiaceae*) (45.8–49.7%) (Supplementary Figures 1A,B). Additionally, there were low abundances of unclassified bacteria (9.4–10.3%) and other families (2.9–5.0%) in all plots (Supplementary Figure 1B). N fertilization increased the relative abundance of *Methylocystaceae* (aCO_2_, from 16.6 to 26.0%; eCO_2_, from 13.8 to 24.7%) while reducing the relative abundance of *Rhizobiaceae* (aCO_2_, from 10.2 to 1.1%; eCO_2_, from 9.5 to 4.3%) under both aCO_2_ and eCO_2_ treatment (Supplementary Figure 1B). Combined with the community compositions at the genus level, the relative abundances of *Methylosinus* (type II methanotrophs) and *Rhizobium* (traditional N_2_-fixing bacteria) were changed under combined of elevated CO_2_, N fertilization treatment. The former showed an increasing trend, while the latter showed a decreasing trend (Figure 4).

**FIGURE 5:**
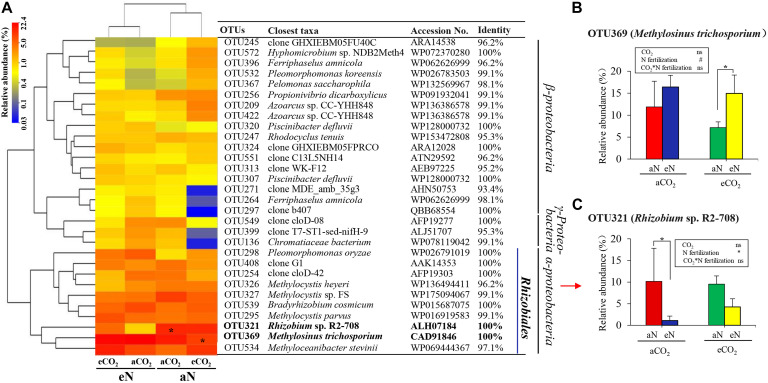
Heatmap analysis of the similarities and differences of N_2_-fixing bacteria of rice roots at the heading stage under elevated CO_2_ and nitrogen fertilization treatments from the top 30 OTUs **(A)**. The color intensity in each cell shows the relative abundance of a genus/species in a sample. Bold typeface indicates the main OTUs **(A)**. Interactive effects of elevated CO_2_ and nitrogen fertilization on the relative abundance of the two main OTUs **(B,C)**. aCO_2_, ambient CO_2_; eCO_2_, elevated atmospheric CO_2_. aN, no N fertilization; eN, elevated N fertilization. Considering the split-plot design, the statistics, the analysis of variance were derived using linear mixed-effects model procedure to test the effect of elevated CO_2_, N fertilization, and their interactions on the relative abundance of the main OTUs at the same growth stage. Multiple comparison were further used to determine which groups were significantly different with Duncan test. ^∗^*p* ≤ 0.05, ^#^ 0.05 < *p* ≤ 0.10, ns *p* > 0.10.

To further explore the differences between species, we compared the total N_2_-fixing bacterial community at the OTU level. The top 30 OTUs were clustered into three main groups, which were affiliated with α*-proteobacteria*, β*-proteobacteria*, and γ*-proteobacteria* (Figure 5A). The two OTUs, OTU 369 and OTU 321, were similar to *Methylosinus trichosporium* (CAD91846) (100% sequence identity) and *Rhizobium* sp. R2-708 (ALH07184) (100% sequence identity), respectively, which were affiliated with α*-proteobacteria, Rhizobiales* (Figure 5A). Linear mixed-effects model analyses showed that nitrogen fertilization had a marginally significant effect on the relative abundance of *Methylosinus trichosporium* (OTU 369) [*F*_(1, 4)_ = 5.014, *p* = 0.089], and had a significant effect *Rhizobium sp.* R2-708 (OTU 321) [*F*_(1, 4)_ = 8.017, *p* = 0.047], while no significant effects of elevated CO_2_ and interaction [CO_2_ × N fertilization] were observed (Figures 5B,C). In multiple comparison, N fertilization significantly increased *Methylosinus trichosporium* under eCO_2_ treatment (the relative abundance from 7.2 to 15.0%) (*p* = 0.040) (Figure 5B) and decreased *Rhizobium sp.* R2-708 (from 10.2 to 1.1%) (*p* = 0.027) under aCO_2_ treatment (Figure 5C). Under eCO_2_ treatment, *Rhizobium* sp. R2-708 decreased with N fertilization (from 9.5 to 4.3%), but the difference was not significant (*p* = 0.156) (Figure 5C).

### C1 Compound Cycle-Related Root-Associated N_2_-Fixing Bacteria at the Heading Stage

Calculating the total relative abundances at the OTU level clearly showed that N_2_-fixing bacteria of the C1 compound cycle, which include mainly methane-oxidizing bacteria (*Methylosinus, Methylocystis*, unclassified *Methylocystis* and *Pleomorphomonas*) and methanol-oxidizing bacteria (*Methyloceanibacter*, *Rhizobium*, and *Bradyrhizobium*), were predominant in rice roots (42.64–49.03%) (Table 1).

**TABLE 1:** Relative abundance (%) of methylotrophs at the genus level in the total N_2_-fixing bacterial communities of rice roots at the heading stage.

		Ambient CO_2_		Elevated atmospheric CO_2_	

	Closest taxa^*a*^	aN	eN	aN	eN
Methane oxidizing type	*Methylosinus trichosporium*	11.88 ± 5.85	16.43 ± 2.64	7.17 ± 1.29	14.94 ± 4.19
	unclassified Methylocystis	11.27 ± 3.56	7.58 ± 11.37	6.48 ± 6.97	4.91 ± 3.72
	*Methylocystis parvus*	3.65 ± 2.73	4.00 ± 1.16	4.21 ± 2.35	5.78 ± 0.79
	*Pleomorphomonas*	0.91 ± 0.55	5.14 ± 6.92	2.24 ± 1.67	3.59 ± 3.11
Methanol oxidizing type	*Methyloceanibacter stevinii*	4.21 ± 1.95	6.55 ± 4.70	8.12 ± 2.46	11.10 ± 3.87
	*Rhizobium*	10.15 ± 7.68	1.10 ± 1.04	9.51 ± 1.92	4.27 ± 1.87
	*Bradyrhizobium*	3.81 ± 0.13	6.25 ± 2.22	4.90 ± 1.07	4.44 ± 1.59
Total relative abundance		45.89	47.05	42.64	49.03

*aN, no N fertilization; eN, elevated N fertilization.*

*^*a*^nifH gene sequences similarity 96–100%.*

## Discussion

We demonstrated the combined effects of elevated CO_2_ and nitrogen fertilization on rice root-associated diazotrophic communities using qPCR and sequencing analysis. *nifH* gene abundance significantly increased with rice growth. The combined effect of eCO_2_ and eN stimulated the abundance of *nifH* genes in rice roots and rhizospheres at the HI stage compared at the TI stage (Figures 2A,B). Moreover, the combined effect of eCO_2_ and eN promoted the relative abundance of root-associated methane-oxidizing bacteria while inhibiting heterotrophic diazotrophs (Figure 5). To the best of our knowledge, this study is the first to analyze the responses of the structure and gene abundance of root-associated diazotrophic communities to elevated CO_2_ and nitrogen fertilization treatment using the functional gene *nifH*.

The abundance of *nifH* genes is positively correlated with the rates of N_2_ fixation (Hsu and Buckley, 2009; Reed et al., 2011). *nifH* gene abundance in the roots and rhizospheres at the HI stage was significantly higher than that of the TI stage throughout all samples, and N fertilization decreased its abundance under aCO_2_ treatment but increased it under eCO_2_ treatment (Figures 2A,B). Plant growth requires more nitrogen at the HI stage than at the TI stage, and that nitrogen is acquired through the enrichment of root-associated nitrogen-fixing bacteria (Hurek and Reinhold-Hurek, 2003). Generally, nitrogen inputs inhibit N_2_ fixation because the nitrogenase encoded by the *nifH* gene is sensitive to nitrogen inputs (Smercina et al., 2019). The negative effect of nitrogen fertilization on N_2_ fixation has been well reported in legumes and other plants under ambient CO_2_ conditions (Reed et al., 2011; Fan et al., 2019), and these results are consistent with those of our study. In addition, a previous study carried out on the same FACE platform also showed that eCO_2_ increased the abundance of the *nifH* gene in soil with nitrogen inputs and cultivation of the same rice cultivar (*Japonica*, WuYunJing) (Yu et al., 2018). Plant growth enhances the N demand due to the greater photosynthetic activity and plant biomass accumulation under elevated CO_2_ (Rogers et al., 2009). For legume plants, eCO_2_ enhanced N_2_ fixation by increasing nodule number and biomass at later growth stages but did not reduce N_2_ fixation (Li et al., 2017). In addition, a ^15^N labeling experiment showed that eCO_2_ reduced the inhibitory effect of nitrogen on the N_2_ fixation activity of soil cultivated with *Pisum sativum* (Butterly et al., 2016). Taken together, these results suggest that the combination of elevated CO_2_ and nitrogen fertilization stimulated *nifH* gene abundance in our study. Other investigations of symbiotic fixation responses to CO_2_ suggest that N_2_ fixation increases only in response to eCO_2_ when nutrients such as P and Mo are added (van Groenigen et al., 2006), and fertilizer often contains P, Mo, and other elements. However, the mechanism of the combined effect of nitrogen fertilization and eCO_2_ on the abundance of the diazotrophic community is complex and requires future in-depth study. In contrast, elevated CO_2_ did not affect the *nifH* gene abundance of roots and rhizospheres under the treatment with no N fertilization at the HI stage in our study (*p* = 0.607, for roots; *p* = 0.109, for rhizosphere soils) (Figure 2). This result is consistent with that previously reported by van Groenigen et al. (2006), who showed using meta-analysis that elevated CO_2_ did not affect N_2_ fixation by legume plants when N fertilizer was not applied. It is likely that rice plants enrich N_2_-fixing bacteria at specific growth stages, and nitrogen fertilizer mainly influences N_2_-fixing bacteria not only in the root zone but also in the bulk soil of paddy rice fields under elevated CO_2_ treatment such as those projected for the future.

Some changes of the root-associated N_2_-fixing bacterial community composition at the HI stage under the combined effect of elevated CO_2_ and N fertilization treatment were observed. *Methylosinus* (*Methylocystaceae*), *Methylocystis* (*Methylocystaceae*), *Rhizobium* (*Rhizobiaceae*), *Methyloceanibacter* (*Rhizobiales*), *Pleomorphomonas* (*Methylocystaceae*), *Bradyrhizobium* (*Bradyrhizobiaceae*) and other unclassified *Rhizobiales* within the order *Rhizobiales* were commonly detected as the main diazotrophs from all root samples. But eCO_2_ increased the relative abundance of *Methylosinus trichosporium* (OTU369) under N fertilization treatment (Figure 5B). One Japanese FACE experiment showed that elevated CO_2_ tended to decrease the relative abundance of *Methylocystis* and *Methylosinus* in rice roots at the ripening stage, which was observed through 16S rRNA gene sequencing; however, statistical analysis was lacking in that experiment (Okubo et al., 2014). The genera *Methylosinus* and *Methylocystis* of the family *Methylocystacea* are methane-oxidizing bacteria that are frequently detected in rice roots and forest soils as diazotrophs (Buckley et al., 2008; Bao et al., 2014a; Shinoda et al., 2019) and in particular as the dominant group in later rice growth stages (Shrestha et al., 2010). Moreover, type II methanotrophs, including *Methylosinus*, can assimilate up to 50% of their biomass from CO_2_ (Trotsenko and Murrell, 2008). These findings can explain the results observed in our study, i.e., the increased relative abundance of *Methylosinus trichosporium* (OTU369) under the combination of eCO_2_ and N fertilization. Thus, the increased *nifH* gene abundance of roots under the combination of eCO_2_ and eN might explain the increasing *nifH* copy numbers of methanotrophs (Figure 2A). Apart from *Methylosinus trichosporium*, N fertilization decreased the relative abundance of *Rhizobium* sp. R2-708 (OTU321) under aCO_2_ treatment (Figure 5C). *Rhizobium* is a well-known N_2_-fixing bacterium that is frequently detected in legumes as well as in rice roots (Zahran, 1999; Bao et al., 2014a; Yoneyama et al., 2019) and other plants. Heterotrophic N_2_-fixing bacteria, including *Rhizobium*, are often inhibited by nitrogen inputs (Kumar et al., 2018). This result showed that compared to *Rhizobium*, root-associated diazotrophic *Methylosinus* was more stable under climate change conditions or nitrogen fertilization, even when combined. In addition, *nifH*-containing microbes are complex and high diversity, which may have a relationship with gene horizontal transfer of *nifH* (Remigi et al., 2016). Horizontal transfer of *nifH* gene is more complicated. Horizontal transfer of *nifH* gene was related to the consistency between the phylogenies of *nifH* gene (or phylogenetic tree of *nifH*, *nifD*, and *nifK*) and 16S rRNA gene (Bolhuis et al., 2010). There was no overall horizontal gene transfer of *nifH* gene across phylogeny in our study (Figure 5A). The main *nifH* sequences of OTUs were closely related to *Methylocystis* and *Methylosinus* of *Methylocystaceae* (type II methanotrophs), *Rhizobium* (*Rhizobiaceae*) and *Bradyrhizobiacea*, and they closed to each other within *Rhizobiales* of *Alphaproteobacteria* (Figure 5A). Therefore, the result is consistent with phylogenetic analysis with 16S rRNA gene (Auman et al., 2001; Dedysh et al., 2004). But both sequences of OTU572 and OTU532 were closely related to *Hyphomicrobium* sp. NDB2 (*nifH* sequence identity, 100%) and *Pleomorphomonas koreensis* (99.1%), respectively, and were clustered into *Betaproteobacteria* (Figure 5A). This result is inconsistent with a previous study showed that both *Hyphomicrobium* and *Pleomorphomonas* belong to the *Rhizobiales* of *Alphaproteobacteria* with the 16S rRNA (Hordt et al., 2020). Thus, the *nifH* of above two OTUs may be acquired through horizontal gene transfer across phylogeny.

Interestingly, most diazotrophs were related to the C1 compound cycle, which includes methane-oxidizing bacteria and methanol-utilizing bacteria, in all root samples in our study (Table 1). In rice paddies, methane-oxidizing bacteria are the most dominant microbes among C1 bacteria, because continuous methane production in anaerobic sediment layers or in roots provides a carbon and energy source for root-associated diazotrophic methanotrophs (Bao et al., 2014a). eCO_2_ can significantly increase methane emissions (Tokida et al., 2011). Usually, methanotrophs are able to fix N_2_ under N-limited conditions (Bodelier and Laanbroek, 2004; Shinoda et al., 2019). However, Zheng et al. (2019) found that N_2_ fixation was stimulated by the substrate C/N ratio rather than by the N concentration alone. N_2_ fixation is enhanced when substrate C is relatively plentiful and substrate N is relatively scarce (Reed et al., 2011). Thus, the availability of substrate C is critically important for N_2_ fixation. eCO_2_ can increase the photosynthetic C fixation capacity and biomass accumulation of rice plants (Rogers et al., 2009), which must take up large amounts of N (Shimono et al., 2009). Elevated N fertilization can help maintain a higher photosynthetic rate and ensure a higher yield under eCO_2_ (Kimball et al., 2002). Therefore, more root-associated diazotrophic methanotrophs may be needed to provide sufficient N_2_ fixation to meet the demand for a higher C/N ratio required for rice biomass accumulation under both eN and eCO_2_ treatments. This may be one explanation for the N_2_ fixation performed by root-associated diazotrophic methanotrophs at high N levels. The N_2_-fixing activity of diazotrophic methanotrophs under N-rich conditions needs to be studied in more detail in the future. Moreover, methanotrophic N_2_-fixing bacteria (*Methylosinus* and *Methylocystis*) in the roots, and other N_2_-fixing bacteria, such as *Bradyrhizobium, Rhizobium*, and *Methyloceanibacter*, which are frequently detected as methanol-oxidizing bacteria using metaproteomics or functional gene *mxaF*/*xoxF* sequencing analysis (Bao et al., 2014a; Macey et al., 2020), may utilize the methanol produced via the methane oxidation process to perform N_2_ fixation. However, this relationship changes with nitrogen supplementation because the N_2_-fixing capacity of *Bradyrhizobium* and *Rhizobium* is inhibited by nitrogen (Kumar et al., 2018), while that of diazotrophic methanotrophs is more stable. In addition, eCO_2_ significantly increases the total organic carbon content of rice root exudates (Bhattacharyya et al., 2013), which may influence the diversity and activity of rhizospheric and root-associated C1 bacteria. Macey et al. (2020) reported that methanol-utilizing methylotrophs were widely distributed in land plant-associated soil because they use the methanol produced as a metabolic byproduct during plant growth. Thus, C1-cycling bacteria in the root zones of both terrestrial plants and aquatic plants are important for the balance of carbon and nitrogen cycling and can increase benefits for plant growth.

## Conclusion

The abundance of the *nifH* gene in rice roots greatly increased at reproductive stages compared to vegetative growth stages. Elevated CO_2_ and N fertilization significantly affect the abundance of the *nifH* gene in rice roots at two growth stages, but the interaction [CO_2_ × N fertilization] only significantly affects *nifH* gene abundance of roots at the heading stage. Most root-associated diazotrophs (which accounted for approximately 50% of the relative abundance) were related to methanotrophs and methylotrophs at the HI stage. Of these, elevated CO_2_ and N fertilization significantly affected the relative abundances of root-associated *Methylosinus* and *Rhizobium*. The results of this study will contribute to expanding our understanding of microorganism-related C/N dynamics in rice fields under future climate change conditions.

## Data Availability Statement

The datasets presented in this study can be found in online repositories. The names of the repository/repositories and accession number(s) can be found in the article/Supplementary Material.

## Author Contributions

ZB and CZ designed the study. JH, WC, and SZ performed the experiments. JL, MZ, YL, ZJ, RY, JZ, and ZB analyzed the data. JL and ZB wrote the manuscript. All authors contributed to the article and approved the submitted version.

## Conflict of Interest

The authors declare that the research was conducted in the absence of any commercial or financial relationships that could be construed as a potential conflict of interest.

## References

[B1] Auman A. J. Speake C. C. Lidstrom M. E. (2001). nifH sequences and nitrogen fixation in type I and type II methanotrophs. *Appl. Environ. Microbiol.* 67 4009–4016. 10.1128/aem.67.9.4009-4016.2001 11525998 PMC93122

[B2] Banger K. Tian H. Lu C. (2012). Do nitrogen fertilizers stimulate or inhibit methane emissions from rice fields? *Glob. Chang. Biol.* 18 3257–3267. 10.1111/j.1365-2486.2012.02762.x 28741830

[B3] Bao Z. H. Okubo T. Kubota K. Kasahara Y. Tsurumaru H. Anda M. (2014a). Metaproteomic identification of diazotrophic methanotrophs and their localization in root tissues of field-grown rice plants. *Appl. Environ. Microbiol.* 80 5043–5052. 10.1128/aem.00969-14 24928870 PMC4135783

[B4] Bao Z. H. Watanabe A. Sasaki K. Okubo T. Tokida T. Liu D. Y. (2014b). A rice gene for microbial symbiosis, Oryza sativa CCaMK, reduces CH_4_ flux in a paddy field with low nitrogen input. *Appl. Environ. Microbiol.* 80 1995–2003. 10.1128/aem.03646-13 24441161 PMC3957643

[B5] Berendsen R. L. Pieterse C. M. Bakker P. A. (2012). The rhizosphere microbiome and plant health. *Trends Plant Sci.* 17 478–486. 10.1016/j.tplants.2012.04.001 22564542

[B6] Berthrong S. T. Yeager C. M. Gallegos-Graves L. Steven B. Eichorst S. A. Jackson R. B. (2014). Nitrogen fertilization has a stronger effect on soil nitrogen-fixing bacterial communities than elevated atmospheric CO_2_. *Appl. Environ. Microbiol.* 80 3103–3112. 10.1128/aem.04034-13 24610855 PMC4018900

[B7] Bhattacharyya P. Roy K. S. Neogi S. Manna M. C. Adhya T. K. Rao K. S. (2013). Influence of elevated carbon dioxide and temperature on belowground carbon allocation and enzyme activities in tropical flooded soil planted with rice. *Environ. Monit. Assess.* 185 8659–8671. 10.1007/s10661-013-3202-7 23612768

[B8] Bodelier P. L. Laanbroek H. J. (2004). Nitrogen as a regulatory factor of methane oxidation in soils and sediments. *FEMS Microbiol. Ecol.* 47 265–277. 10.1016/s0168-6496(03)00304-0 19712315

[B9] Bolhuis H. Severin I. Confurius-Guns V. Wollenzien U. I. A. Stal L. J. (2010). Horizontal transfer of the nitrogen fixation gene cluster in the cyanobacterium *Microcoleus chthonoplastes*. *ISME J.* 4 121–130. 10.1038/ismej.2009.99 19741736

[B10] Bosse U. Frenzel P. (1997). Activity and distribution of methane-oxidizing bacteria in flooded rice soil microcosms and in rice plants (*Oryza sativa*). *Appl. Environ. Microbiol.* 63 1199–1207. 10.1128/aem.63.4.1199-1207.1997 16535562 PMC1389540

[B11] Buckley D. H. Huangyutitham V. Hsu S. F. Nelson T. A. (2008). N15-DNA-stable isotope probing of diazotrophic methanotrophs in soil. *Soil Biol. Biochem.* 40 1272–1283. 10.1016/j.soilbio.2007.05.006

[B12] Butterly C. R. Armstrong R. Chen D. L. Tang C. X. (2016). Free-air CO_2_ enrichment (FACE) reduces the inhibitory effect of soil nitrate on N2 fixation of *Pisum sativum*. *Ann. Bot.* 117 177–185. 10.1093/aob/mcv140 26346721 PMC4701144

[B13] Caporaso J. G. Kuczynski J. Stombaugh J. Bittinger K. Bushman F. D. Costello E. K. (2010). QIIME allows analysis of high-throughput community sequencing data. *Nat. Methods* 7 335–336. 20383131 10.1038/nmeth.f.303 PMC3156573

[B14] Chaparro J. M. Badri D. V. Vivanco J. M. (2014). Rhizosphere microbiome assemblage is affected by plant development. *ISME J.* 8 790–803. 10.1038/ismej.2013.196 24196324 PMC3960538

[B15] Das S. Adhya T. K. (2012). Dynamics of methanogenesis and methanotrophy in tropical paddy soils as influenced by elevated CO_2_ and temperature interaction. *Soil Biol. Biochem.* 47 36–45. 10.1016/j.soilbio.2011.11.020

[B16] Dedysh S. N. Ricke P. Liesack W. (2004). NifH and NifD phylogenies: an evolutionary basis for understanding nitrogen fixation capabilities of methanotrophic bacteria. *Microbiology* 150 1301–1313. 10.1099/mic.0.26585-0 15133093

[B17] Edgar R. C. (2010). Search and clustering orders of magnitude faster than BLAST. *Bioinformatics* 26 2460–2461. 10.1093/bioinformatics/btq461 20709691

[B18] Eller G. Frenzel P. (2001). Changes in activity and community structure of methane-oxidizing bacteria over the growth period of rice. *Appl. Environ. Microbiol.* 67 2395–2403. 10.1128/aem.67.6.2395-2403.2001 11375143 PMC92887

[B19] Fan K. Delgado-Baquerizo M. Guo X. Wang D. Wu Y. Zhu M. (2019). Suppressed N fixation and diazotrophs after four decades of fertilization. *Microbiome* 7:143. 10.1186/s40168-019-0757-8 PMC6824023 31672173

[B20] Food and Agriculture Organization of the United Nations (2002). *Concern About Rice Production Practices.* Rome: Food and Agriculture Organization of the United Nations.

[B21] Gaby J. C. Buckley D. H. (2014). A comprehensive aligned nifH gene database: a multipurpose tool for studies of nitrogen-fixing bacteria. *Database* 8:bau001. 10.1093/database/bau001 PMC3915025 24501396

[B22] García-Palacios P. Vandegehuchte M. L. Shaw E. A. Dam M. Post K. H. Ramirez K. S. (2015). Are there links between responses of soil microbes and ecosystem functioning to elevated CO_2_, N deposition and warming? a global perspective. *Glob. Chang. Biol.* 21 1590–1600. 10.1111/gcb.12788 25363131

[B23] Heim A. Moser N. Blum H. Schmidt M. W. I. (2009). How far do experimentally elevated CO_2_ levels reach into the surrounding? an example using the 13C label of soil organic matter as an archive. *Glob. Chang. Biol.* 15 1598–1602. 10.1111/j.1365-2486.2009.01843.x

[B24] Hordt A. Lopez M. G. Meier-Kolthoff J. P. Schleuning M. Weinhold L. M. Tindall B. J. (2020). Analysis of 1,000+ type-strain genomes substantially improves taxonomic classification of Alphaproteobacteria. *Front. Microbiol.* 11:468. 10.3389/fmicb.2020.00468 PMC7179689 32373076

[B25] Hsu S. F. Buckley D. H. (2009). Evidence for the functional significance of diazotroph community structure in soil. *ISME J.* 3 124–136. 10.1038/ismej.2008.82 18769458

[B26] Hurek T. Reinhold-Hurek B. (2003). Azoarcus sp. strain bh72 as a model for nitrogen-fixing grass endophytes. *J. Biotechnol.* 106 169–178. 10.1016/j.jbiotec.2003.07.010 14651859

[B27] Ikeda S. Sasaki K. Okubo T. Yamashita A. Terasawa K. Bao Z. H. (2014). Low nitrogen fertilization adapts rice root microbiome to low nutrient environment by changing biogeochemical functions. *Microbes Environ.* 29 50–59. 10.1264/jsme2.me13110 24463575 PMC4041235

[B28] IPCC (2013). “Evaluation of climate models,” in *Climate Change: the Physical Science Basis. Contribution of Working Group I to the Fifth Assessment Report of the Intergovernmental Panel on Climate Change*, eds Stocker T. F. Qin D. Plattner G. K. Tignor M. Allen S. K. Boschung J. (Cambridge: Cambridge University Press).

[B29] Ishii S. Ikeda S. Minamisawa K. Senoo K. (2011). Nitrogen cycling in rice paddy environments: past achievements and future challenges. *Microbes Environ.* 26 282–292. 10.1264/jsme2.me11293 22008507

[B30] Ju X. T. Xing G. X. Chen X. P. Zhang S. L. Zhang L. J. Liu X. J. (2009). Reducing environmental risk by improving N management in intensive Chinese agricultural systems. *Proc. Natl. Acad. Sci. U.S.A.* 106 3041–3046. 10.1073/pnas.0813417106 19223587 PMC2644255

[B31] Kimball B. A. Kobayashi K. Bindi M. (2002). Responses of agricultural crops to free-air CO_2_ enrichment. *Adv. Agron* 77 293–368. 10.1016/s0065-2113(02)77017-x 12557686

[B32] Kimura M. (2004). “Counting and isolation of rhizosphere microorganism. in: Japanese society of soil microbiology,” in *Experimental Methods in Soil Microbiology*, 3rd Edn, ed. Japanese society of soil microbiology. (Tokyo: Yokendo Co., Ltd).

[B33] Kumar U. Nayak A. K. Shahid M. Gupta V. V. S. R. Panneerselvam P. Mohanty S. (2018). Continuous application of inorganic and organic fertilizers over 47 years in paddy soil alters the bacterial community structure and its influence on rice production. *Agric. Ecosyst. Environ.* 262 65–75. 10.1016/j.agee.2018.04.016

[B34] Li Y. S. Yu Z. H. Liu X. B. Mathesius U. Wang G. H. Tang C. X. (2017). Elevated CO_2_ increases nitrogen fixation at the reproductive phase contributing to various yield responses of soybean cultivars. *Front. Plant Sci.* 8:1546. 10.3389/fpls.2017.01546 PMC5603704 28959266

[B35] Luo Y. Su B. Currie W. S. Dukes J. S. Finzi A. Hartwig U. (2004). Progressive nitrogen limitation of ecosystem responses to rising atmospheric carbon dioxide. *Bioscience* 54 731–739. 10.1641/0006-3568(2004)054[0731:pnloer]2.0.co;2

[B36] Macey M. C. Pratscher J. Crombie A. T. Murrell J. C. (2020). Impact of plants on the diversity and activity of methylotrophs in soil. *Microbiome* 8:31. 10.1186/s40168-020-00801-4 PMC7065363 32156318

[B37] Nouchi I. Mariko S. Aoki K. (1990). Mechanism of methane transport from the rhizosphere to the atmosphere through rice plants. *Plant Physiol.* 94 59–66. 10.1104/pp.94.1.59 16667719 PMC1077189

[B38] Okubo T. Tokida T. Ikeda S. Bao Z. H. Tago K. Hayatsu M. (2014). Effects of elevated carbon dioxide, elevated temperature, and rice growth stage on the community structure of rice root–associated bacteria. *Microbes Environ.* 29 184–190. 10.1264/jsme2.me14011 24882221 PMC4103525

[B39] Poly F. Monrozier L. J. Bally R. (2001). Improvement in the RFLP procedure for studying the diversity of nifH genes in communities of nitrogen fixers in soil. *Res. Microbiol.* 152 95–103. 10.1016/s0923-2508(00)01172-4 11281330

[B40] Reed S. C. Cleveland C. C. Townsend A. R. (2011). Functional ecology of free-living nitrogen-fixation: a contemporary perspective. *Annu. Rev. Ecol. Evol. Syst.* 42 489–512. 10.1146/annurev-ecolsys-102710-145034

[B41] Remigi P. Zhu J. Young J. P. W. Masson C. B. (2016). Symbiosis within symbiosis: evolving nitrogen-fixing legume symbionts. *Trends Microbiol.* 24 63–75. 10.1016/j.tim.2015.10.007 26612499

[B42] Rogers A. Ainsworth E. A. Leakey A. D. B. (2009). Will elevated carbon dioxide concentration amplify the benefits of nitrogen fixation in legumes? *Plant Physiol.* 151 1009–1016. 10.1104/pp.109.144113 19755541 PMC2773101

[B43] Schloss P. D. Westcott S. L. Ryabin T. Hall J. R. Hartmann M. Hollister E. B. (2009). Introducing mothur: open-source, platform-independent, community-supported software for describing and comparing microbial communities. *Appl. Environ. Microbiol.* 75 7537–7541. 10.1128/aem.01541-09 19801464 PMC2786419

[B44] Shimono H. Okada M. Yamakawa Y. Nakamura H. Kobayashi K. Hasegawa T. (2009). Genotypic variation in rice yield enhancement by elevated CO_2_ relates to growth before heading and not to maturity group. *J. Exp. Bot.* 60 523–532. 10.1093/jxb/ern288 19050063 PMC2651455

[B45] Shinoda R. Bao Z. H. Minamisawa K. (2019). CH_4_ oxidation-dependent 15N2 fixation in rice roots in a low-nitrogen paddy field and in Methylosinus sp. strain 3S-1 isolated from the roots. *Soil Biol. Biochem.* 132 40–46. 10.1016/j.soilbio.2019.01.021

[B46] Shrestha M. Shrestha P. M. Frenzel P. Conrad R. (2010). Effect of nitrogen fertilization on methane oxidation, abundance, community structure, and gene expression of methanotrophs in the rice rhizosphere. *ISME J.* 4 1545–1556. 10.1038/ismej.2010.89 20596069

[B47] Smercina D. N. Evans S. E. Friesen M. L. Tiemann L. K. (2019). To fix or not to fix: controls on free-living nitrogen fixation in the rhizosphere. *Appl. Environ. Microbiol.* 85:e02546-18. 10.1128/AEM.02546-18 PMC6414387 30658971

[B48] Tamura K. Peterson D. Peterson N. Stecher G. Nei M. Kumar S. (2011). MEGA5: molecular evolutionary genetics analysis using maximum likelihood, evolutionary distance, and maximum parsimony methods. *Mol. Biol. Evol.* 28 2731–2739. 10.1093/molbev/msr121 21546353 PMC3203626

[B49] Thompson J. D. Higgins D. G. Gibson T. J. (1994). CLUSTAL W: improving the sensitivity of progressive multiple sequence alignment through sequence weighting, positions-specific gap penalties and weigh matrix choice. *Nucleic Acids Res.* 22 4673–4680. 10.1093/nar/22.22.4673 7984417 PMC308517

[B50] Tokida T. Adachi M. Cheng W. G. Nakajima Y. Fumoto T. Matsushima M. (2011). Methane and soil CO_2_ production from current-season photosynthates in a rice paddy exposed to elevated CO_2_ concentration and soil temperature. *Glob. Chang. Biol.* 17 3327–3337. 10.1111/j.1365-2486.2011.02475.x

[B51] Trotsenko Y. A. Murrell J. C. (2008). Metabolic aspects of aerobic obligate methanotrophy. *Adv. Appl. Microbiol.* 63 183–229. 10.1016/s0065-2164(07)00005-6 18395128

[B52] Usui Y. Sakai H. Tokida T. Nakamura H. Nakagawa H. Hasegawa T. (2014). Heat-tolerant rice cultivars retain grain appearance quality under free-air CO_2_ enrichment. *Rice* 7:6. 10.1186/s12284-014-0006-5 PMC4052674 24920972

[B53] van Groenigen K. J. Six J. Hungate B. A. de Graaff M. A. van Breemen N. van Kessel C. (2006). Element interactions limit soil carbon storage. *Proc. Natl. Acad. Sci. U.S.A.* 103 6571–6574. 10.1073/pnas.0509038103 16614072 PMC1458924

[B54] Wang Q. Quensen J. F. Fish J. A. Lee T. K. Sun Y. Tiedje J. M. (2013). Ecological patterns of nifH genes in four terrestrial climatic zones explored with targeted metagenomics using FrameBot, a new informatics tool. *MBio* 4:e00592-13. 10.1128/mBio.00592-13 PMC3781835 24045641

[B55] Wartiainen I. Eriksson T. Zheng W. W. Rasmussen U. (2008). Variation in the active diazotrophic community in rice paddy—nifH PCR-DGGE analysis of rhizosphere and bulk soil. *Appl. Soil Ecol.* 39 65–75. 10.1016/j.apsoil.2007.11.008

[B56] Xie B. H. Zhou Z. X. Mei B. L. Zheng X. H. Dong H. B. Wang R. (2012). Influences of free-air CO_2_ enrichment (FACE), nitrogen fertilizer and crop residue incorporation on CH_4_ emissions from irrigated rice fields. *Nutr. Cycl. Agroecosyst.* 93 373–385. 10.1007/s10705-012-9523-z

[B57] Yang L. X. Huang J. Y. Yang H. J. Zhu J. G. Liu H. J. Dong G. C. (2006). The impact of free-air CO_2_ enrichment (FACE) and N supply on yield formation of rice crops with large panicle. *Field Crop Res.* 98 141–150. 10.1016/j.fcr.2005.12.014

[B58] Yoneyama T. Terakado-Tonooka J. Bao Z. H. Minamisawa K. (2019). Molecular analyses of the distribution and function of diazotrophic rhizobia and methanotrophs in the tissues and rhizosphere of non-leguminous plants. *Plants* 8:408. 10.3390/plants8100408 31614562 PMC6843303

[B59] Yu Y. J. Zhang J. W. Petropoulos E. Baluja M. Q. Zhu C. W. Zhu J. G. (2018). Divergent responses of the diazotrophic microbiome to elevated CO_2_ in two rice cultivars. *Front. Microbiol.* 9:1139. 10.3389/fmicb.2018.01139 PMC5992744 29910783

[B60] Zahran H. H. (1999). Rhizobium-legume symbiosis and nitrogen fixation under severe conditions and in an arid climate. *Microbiol. Mol. Biol. R.* 63 968–989. 10.1128/mmbr.63.4.968-989.1999 PMC98982 10585971

[B61] Zheng M. H. Chen H. Li D. J. Luo Y. Q. Mo J. M. (2019). Substrate stoichiometry determines nitrogen fixation throughout succession in southern Chinese forests. *Ecol. Lett.* 23 336–347. 10.1111/ele.13437 31802606

[B62] Zhu C. W. Xu X. Wang D. Zhu J. G. Liu G. Q. (2015). An indica rice genotype showed a similar yield enhancement to that of hybrid rice under free air carbon dioxide enrichment. *Sci. Rep.* 5:12719. 10.1038/srep12719 PMC4521144 26228872

[B63] Zhu C. W. Xu X. Wang D. Zhu J. G. Liu G. Seneweera S. (2016). Elevated atmospheric [CO_2_] stimulates sugar accumulation and cellulose degradation rates of rice straw. *GCB Bioenergy* 8 579–587. 10.1111/gcbb.12277

