# Supplementary figures and images for: Elevated Atmospheric CO2 and Nitrogen Fertilization Affect the Abundance and Community Structure of Rice Root-Associated Nitrogen-Fixing Bacteria
Source: Front Microbiol. 2021 Apr 21;12:628108. doi: 10.3389/fmicb.2021.628108 (PMC8103900; doi:10.3389/fmicb.2021.628108)

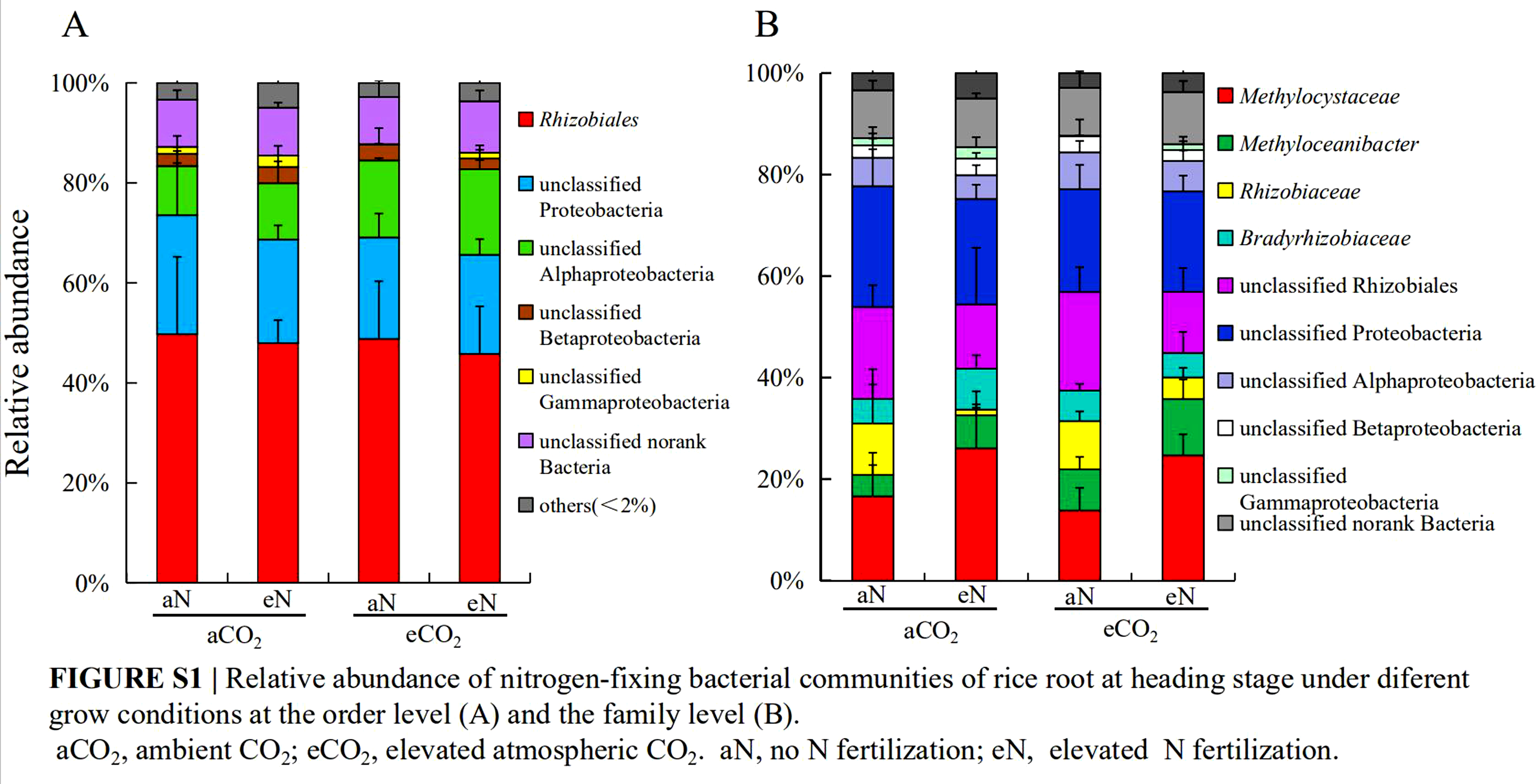

Supplement: Supplementary file 1 [file Image_1.jpg]
