# Supplementary material for: Elevated Atmospheric CO2 and Nitrogen Fertilization Affect the Abundance and Community Structure of Rice Root-Associated Nitrogen-Fixing Bacteria
Source: Front Microbiol. 2021 Apr 21;12:628108. doi: 10.3389/fmicb.2021.628108 (PMC8103900; doi:10.3389/fmicb.2021.628108)
Supplement: Supplementary file 2 [file Table_1.DOCX]

**TABLE S1 |** Environmental data for sampling site (latitude, 32°35'5″N; longitude, 119°42'0″E)

|  | **Rice paddy plots** | | | |
| --- | --- | --- | --- | --- |
| **Environmental data** | **aCO_2_-aN** | **aCO_2_-eN** | **eCO_2_-aN** | **eCO_2_-eN** |
| Chemical properties ^a^ |  |  |  |  |
| TC | 18.4±0.60 | 17.9±0.30 | 18.6±1.56 | 18.5±1.91 |
| TN | 1.6±0.10 | 1.7±0.20 | 2.01±0.10 | 2.07±0.15 |
| TP | / | 0.6±0.03 | / | 0.7±0.01 |
| TK | / | 13.8±0.4 | / | 14.3±0.8 |
| Available P | / | 8.6±2.8 | / | 11.7±1.8 |
| Available K | / | 66.0±8.5 | / | 75.0±8.0 |
| Soil texture (sandy loam) |  |  |  |  |
| Clay (＜0.001 mm) | 13.7% | | | |
| Silt (0.05-0.001 mm) | 28.5% | | | |
| Sand (1-0.05 mm) | 57.8% | | | |
| CO2 (μ mol·mol^−1^) | 400±10 | | 590± 40 | |
| No nitrogen fertilization (kg·ha^-1^·yr^-1^) | 0 | / | 0 | / |
| Elevated nitrogen fertilization (kg·ha^-1^·yr^-1^) | / | 225 | / | 225 |

^a^: g kg^-1^ for SOC, TN and TP; mg kg^-1^ for Available P, Available K.

SOC, soil organic carbon; TN, total nitrogen; TP, total phosphorus; TK, total potassium; Available P, available phosphorus; Available K, available potassium.

aCO_2_, ambient CO_2_; eCO_2_, elevated atmospheric CO_2_. aN, no N fertilization; eN, elevated N fertilization.
