# Supplementary material for: Elevated Atmospheric CO2 and Nitrogen Fertilization Affect the Abundance and Community Structure of Rice Root-Associated Nitrogen-Fixing Bacteria
Source: Front Microbiol. 2021 Apr 21;12:628108. doi: 10.3389/fmicb.2021.628108 (PMC8103900; doi:10.3389/fmicb.2021.628108)
Supplement: Supplementary file 3 [file Table_2.docx]

**TABLE S2 |** The comparison of *nifH* copy numbers at the tillering and heading stages from the same plot with paired samples t test

|  |  |  | Paired Differences^a^ | |  |  |  |
| --- | --- | --- | --- | --- | --- | --- | --- |
| CO_2_ level | N fertilization level | samples | Mean | Std. Deviation | *t* | df | Sig. (2-tailed)^b^ |
| aCO_2_ | aN | roots | 1.32E+09 | 2.19E+08 | 10.462 | 2 | 0.009 |
| aCO_2_ | eN | roots | 3.28E+08 | 3.01E+07 | 18.862 | 2 | 0.003 |
| eCO_2_ | aN | roots | 1.33E+09 | 1.51E+08 | 15.225 | 2 | 0.004 |
| eCO_2_ | eN | roots | 1.77E+09 | 9.79E+07 | 31.343 | 2 | 0.001 |
| aCO_2_ | aN | rhizosphere soils | 1.67E+08 | 3.45E+07 | 8.401 | 2 | 0.014 |
| aCO_2_ | eN | rhizosphere soils | -1.50E+08 | 3.38E+07 | -7.684 | 2 | 0.017 |
| eCO_2_ | aN | rhizosphere soils | 9.67E+07 | 3.82E+07 | 4.381 | 2 | 0.048 |
| eCO_2_ | eN | rhizosphere soils | 8.80E+07 | 1.35E+07 | 11.329 | 2 | 0.008 |

aCO_2_, ambient CO_2_; eCO_2_, elevated atmospheric CO_2_. aN, no N fertilization; eN, elevated N fertilization.

^a^ *nifH* copy numbers at the heading stage minus those at the tillering stage.

^b^ *nifH* copy numbers of both roots and rhizosphere soils at the heading stages were significantly higher than those at the tillering stage (*p* < 0.05).
