# Supplementary material for: Elevated Atmospheric CO2 and Nitrogen Fertilization Affect the Abundance and Community Structure of Rice Root-Associated Nitrogen-Fixing Bacteria
Source: Front Microbiol. 2021 Apr 21;12:628108. doi: 10.3389/fmicb.2021.628108 (PMC8103900; doi:10.3389/fmicb.2021.628108)
Supplement: Supplementary file 4 [file Table_3.docx]

**TABLE S3 | Diversity statistics** **at different sequence similarity levels and the effect of elevated CO_2_, N fertilization, and their interaction on alpha diversity indices (OTU numbers, Shannon and 1/Simpson index) of rice roots at the heading stage ***

| **OTU**  **similarity (%)** | CO_2_ level | N fertilization  level | **No. of**  **sequences** | **No. of OTUs** | **1/D**  **(1/simpson)** | **Shannon** | **Coverage** |
| --- | --- | --- | --- | --- | --- | --- | --- |
| 90 | aCO_2_ | aN | 13373±3915 | 177±16 | 14.71±6.39 | 3.28±0.34 | 0.997±0.001 |
|  | aCO_2_ | eN | 11344±1806 | 148±36 | 11.77±2.47 | 3.17±0.35 | 0.997±0.001 |
|  | eCO_2_ | aN | 13429±1378 | 154±16 | 15.37±3.81 | 3.20±0.23 | 0.997±0.000 |
|  | eCO_2_ | eN | 11899±3264 | 160±27 | 11.14±1.02 | 3.18±0.24 | 0.997±0.002 |
|  | CO_2_ |  |  | *F*_(1, 2)_ = 0.503, *p* = 0.552 | *F*_(1, 2)_ = 0.001, *p* = 0.984 | *F*_(1, 2)_ = 0.798, *p* = 0.466 |  |
|  | N fertilization |  |  | *F*_(1, 4)_ = 0.377, *p* = 0.572 | *F*_(1, 4)_ = 2.320, *p* = 0.202 | *F*_(1, 4)_ = 0.187, *p* = 0.688 |  |
|  | CO_2*_N fertilization |  |  | *F*_(1, 4)_ = 0.801, *p* = 0.421 | *F*_(1, 4)_ = 0.125, *p* = 0.742 | *F*_(1, 4)_ = 0.077, *p* = 0.796 |  |
|  |  |  |  |  |  |  |  |
| 94 | aCO_2_ | aN | 12890±3840 | 348±15 | 20.66±9.24 | 3.86±0.32 | 0.993±0.002 |
|  | aCO_2_ | eN | 10998±1907 | 317±53 | 17.61±3.06 | 3.79±0.34 | 0.993±0.002 |
|  | eCO_2_ | aN | 12594±1333 | 328±31 | 24.97±7.07 | 3.90±0.30 | 0.993±0.001 |
|  | eCO_2_ | eN | 11367±2932 | 327±35 | 18.96±1.66 | 3.88±0.23 | 0.993±0.003 |
|  | CO_2_ |  |  | *F*_(1, 2)_ = 0.054, *p* = 0.839 | *F*_(1, 2)_ = 1.362, *p* = 0.363 | *F*_(1, 2)_ = 0.249, *p* = 0.667 |  |
|  | N fertilization |  |  | *F*_(1, 4)_ = 0.834, *p* = 0.413 | *F*_(1, 4)_ = 1.878, *p* = 0.242 | *F*_(1, 4)_ = 0.065, *p* = 0.812 |  |
|  | CO_2*_N fertilization |  |  | *F*_(1, 4)_ = 0.733, *p* = 0.440 | *F*_(1, 4)_ = 0.200, *p* = 0.678 | *F*_(1, 4)_ = 0.024, *p* = 0.883 |  |
|  |  |  |  |  |  |  |  |
| 99 | aCO_2_ | aN | 10818±3383 | 755±29 | 53.09±18.19 | 5.01±0.18 | 0.975±0.008 |
|  | aCO_2_ | eN | 9313±1860 | 691±143 | 43.87±9.62 | 4.80±0.29 | 0.974±0.006 |
|  | eCO_2_ | aN | 10254±1256 | 735±112 | 61.87±8.55 | 5.05±0.22 | 0.976±0.002 |
|  | eCO_2_ | eN | 9146±2422 | 697±43 | 51.20±9.13 | 4.92±0.13 | 0.971±0.012 |
|  | CO_2_ |  |  | F_(1, 2)_ = 0.015, *p* = 0.915 | F_(1, 2)_ = 1.834, *p* = 0.308 | F_(1, 2)_ = 0.873, *p* = 0.449 |  |
|  | N fertilization |  |  | F_(1, 4)_ = 0.612, *p* = 0.478 | F_(1, 4)_ = 3.703, *p* = 0.127 | F_(1, 4)_ = 1.023, *p* = 0.369 |  |
|  | CO_2*_N fertilization |  |  | F_(1, 4)_ = 0.042, *p* = 0.847 | F_(1, 4)_ = 0.112, *p* = 0.755 | F_(1, 4)_ = 0.066, *p* = 0.810 |  |

aCO_2_, ambient CO_2_; eCO_2_, elevated atmospheric CO_2_. aN, no N fertilization; eN, elevated N fertilization.

*** Considering the split-plot design, the statistics were derived using linear mixed-effects model procedure to test the effect of eCO_2_, N fertilization, and their interactions on alpha diversity indices (OTU numbers, Shannon and 1/Simpson index) at the heading stage.**

**No significant effect was found (*p* > 0.05).**
